# Association of Caucasian-Identified Variants with Colorectal Cancer Risk in Singapore Chinese

**DOI:** 10.1371/journal.pone.0042407

**Published:** 2012-08-03

**Authors:** Lai Fun Thean, Hui Hua Li, Yik Ying Teo, Woon-Puay Koh, Jian-Min Yuan, Mei Lin Teoh, Poh Koon Koh, Choong Leong Tang, Peh Yean Cheah

**Affiliations:** 1 Department of Colorectal Surgery, Singapore General Hospital, Singapore; 2 Department of Clinical Research, Singapore General Hospital, Singapore, Singapore; 3 Saw Swee Hock School of Public Health, National University of Singapore, Singapore; 4 University of Pittsburgh Cancer Institute, and Department of Epidemiology, Graduate School of Public Health, University of Pittsburgh, Pittsburgh, Pennsylvania, United States of America; 5 Health Screening Unit, Singapore General Hospital, Singapore Singapore; 6 Duke-National University of Singapore, Graduate Medical School, National University of Singapore, Singapore; Ohio State University Medical Center, United States of America

## Abstract

**Background:**

Genome-wide association studies (GWAS) in Caucasians have identified fourteen index single nucleotide polymorphisms (iSNPs) that influence colorectal cancer (CRC) risk.

**Methods:**

We investigated the role of eleven iSNPs or surrogate SNPs (sSNPs), in high linkage disequilibrium (LD, r^2^≥0.8) and within 100 kb vicinity of iSNPs, in 2,000 age- and gender-matched Singapore Chinese (SCH) cases and controls.

**Results:**

Only iSNP rs6983267 at 8q24.21 and sSNPs rs6695584, rs11986063, rs3087967, rs2059254, and rs7226855 at 1q41, 8q23.3, 11q23.1, 16q22.1 and 18q21.1 respectively showed evidence of association with CRC risk, with odds ratios (OR) ranging from 1.13 to 1.40. sSNP rs827401 at 10p14 was associated with rectal cancer risk (OR = 0.74, 95% CI 0.63–0.88) but not disease prognosis (OR = 0.91, 95% CI 0.69–1.20). Interestingly, sSNP rs3087967 at 11q23.1 was associated with CRC risk in men (OR = 1.34, 95% CI 1.14–1.58) but not women (OR = 1.07, 95% CI: 0.88–1.29), suggesting a gender-specific role. Half of the Caucasian-identified variants, including the recently fine-mapped BMP pathway loci, *BMP4*, *GREM1*, *BMP2* and *LAMA 5*, did not show any evidence for association with CRC in SCH (OR ∼1; p-value >0.1). Comparing the results of this study with that of the Northern and Hong Kong Chinese, only variants at chromosomes 8q24.21, 10p14, 11q23.1 and 18q21.1 were replicated in at least two out of the three Chinese studies.

**Conclusions:**

The contrasting results between Caucasians and Chinese could be due to different LD patterns and allelic frequencies or genetic heterogeneity. The results suggest that additional common variants contributing to CRC predisposition remained to be identified.

## Introduction

To date, GWAS in the Caucasian populations have uncovered fourteen iSNPs at chromosomes 1q41, 3q26.2, 8q23.3, 8q24.21, 10p14, 11q23.1, 12q13.13, 14q22, 15q13.3, 16q22.1, 18q21.1, 19q13.1, 20p12.3 and 20q13.33 associated with CRC risk [1]. Fine mapping at several of these candidate regions have identified other SNPs that could potentially be functional variants [2], [3]. Since there are significant differences in allelic frequencies and LD patterns across different populations, these variants have to be replicated to ascertain their role in CRC. More than one-third of these variants, for example, were found to have odds ratios in the opposite direction in the African Americans [4].

Only five variants, rs6983267 (8q24.21), rs10795668 (10p14), rs3802842 (11q23.1), rs4939827 (18q21.1) and rs961253 (20p12.3) were replicated in Northern Chinese [5]. More recently, four variants, rs7014346 (8q24.21), rs4779584 (15q13.3), rs10795668 (10p14) and rs4939827 (18q21.1) were replicated in Hong Kong Chinese [6]. There is neither LD nor population structure information in either study. Several reports indicated that there is a ‘north-south’ population structure closely correlated to geographic location and that the greatest genetic difference is between the Northern Han and Southern Han Chinese [7], [8].

We performed genome-wide genotyping on 2,000 age- and gender-matched case-control series of Singapore Chinese (SCH) patients from a single center and population-based healthy controls. The SCH aged 50 years or more comprises mainly descendants of immigrants from the Southern Chinese provinces of Guangdong and Fujian, and is thus representative of the Southern Han Chinese. Determining the genetic risk for CRC in SCH is pertinent as the SCH has the highest CRC incidence amongst all races in Singapore; internationally, its incidence is higher than that of the residents of Shanghai, China and comparable to that of the Caucasian Whites [9].

**Table 1:** Distribution of clinicopathological features among cases and controls.

	Cases	Controls
	(*n* = 991)	(*n* = 993)
	No. (%)	No. (%)
**Sex**		
Male	565 (57.0)	566 (57.0)
Female	426 (43.0)	427 (43.0)
**Age (y)**		
50–60	177 (17.9)	178 (17.9)
61–70	355 (35.8)	366 (36.9)
71–80	336 (33.9)	354 (35.7)
81–90	114 (11.5)	92 (9.3)
91–100	9 (0.9)	2 (0.2)
Median	70	69
**Site of tumour**		
Colon	628 (63.4)	
Rectum	363 (36.6)	
**Duke stage**		
A	98 (9.9)	
B	366 (36.9)	
C	365 (36.8)	
D	154 (15.5)	
Unknown	8 (0.8)	
**Tumour Differentiation**		
Well	93 (9.4)	
Moderate	813 (82.0)	
Poor	56 (5.7)	
Unknown	29 (2.9)	

## Materials and Methods

### Ethics Statement

Collection of samples and clinico-pathological information from patients and controls was undertaken with written informed consent and approval from SingHealth Centralized Institutional Review Board B.

### Sample Collection

Matched specimens of mucosa and tumor are routinely collected and archived from patients undergoing resection at Singapore General Hospital (SGH). SGH is the premier public hospital which treats about half of the CRC patients in Singapore. The matched mucosa specimens collected are typically at least 10 cm away from tumor site. Mucosa specimens from 1,000 sporadic Chinese CRC patients (defined as age 50 or more at date of operation and without dominant family history of FAP and HNPCC) archived over the past ten years were selected as cases for the study.

**Figure 1:**
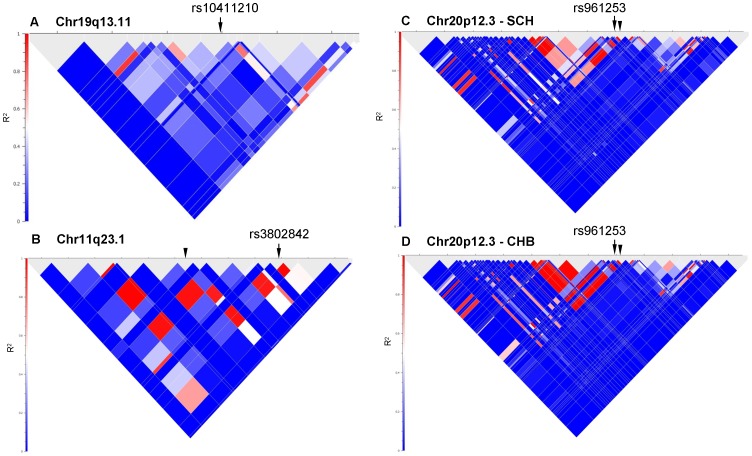
Local pairwise LD plots. The plots for SNPs at chromosomes 19q13.11 (A), 11q23.1 (B), 20p12.3 (C) and 20p12.3 (D) were derived from SCH controls and HapMap CHB individuals respectively. Arrow and arrowhead indicate positions of iSNP and sSNPs interrogated. The sSNPs interrogated at chromosomes 11q23.1 (B) and 20p12.3 (D) were rs3087967 and rs5005940 respectively. LD was measured as R^2^.

Blood samples from 1,000 age- and gender-matched healthy donors from the Singapore Chinese Health Study (SCHS) (n = 931) [10] and the SGH Health Screening Unit (n = 69) constituted the controls of the study. Age was matched to within three years of the year of operation of the cases. The controls were interviewed to ensure that they have no CRC family history.

### Genome-wide Genotyping

Samples were randomized so that consecutively procured samples were not extracted consecutively. Genomic DNA was extracted using standard procedures (Methods S1). Whole-genome scan was performed with Affymetrix GeneChip Human Mapping SNP Array 6.0 consisting 906, 600 SNPs. A 600 ng of genomic DNA sample was digested with the restriction enzymes NspI and StyI, amplified, fragmented, labelled and hybridised to the Array for 16 h as per the manufacturer’s instructions (Affymetrix, Santa Clara, CA). Arrays were scanned with the Affymetrix 3000-7G scanner and analysed with the Genotyping Console v3. CHP files were generated with the Birdseed algorithm. To minimize batch effect, the genotyping was performed by one operator; and matched cases and control specimens were processed and arrayed together.

**Table 2:** Association of CEU-identified iSNP/sSNPs with CRC risk in SCH.

Chr	Gene Symbol	rsID*	MAF^1^(CEU/CHB)	MAF^2^	Regression P	O.R. (95% CI)
				(SCH-control	(Additive Model)	
1q41	DUSP10	rs6695584	0.21/0.20	0.17	0.0864	1.16 (0.98, 1.36)
		**rs6687758**	0.21/0.20			
3q26.2	TERC	rs12638862	0.27/0.58	0.55	0.7946	0.98 (0.87, 1.12)
	MECOM					
	LRRC34	**rs10936599**	0.27/0.57			
8q23.3	EIF3H	**rs16892766**	0.11/0.004	0.0005		
	UTP23	*rs11986063*	0.12/0.06	0.04	0.0262	1.40 (1.04, 1.89)
8q24.21	POU5F1B	**rs6983267**	0.49/0.39	0.44	0.042	1.15 (1.00, 1.31)
10p14	GATA3	rs827401	0.31/0.39	0.47	0.1383	0.91 (0.81, 1.03)
		**rs10795668**	0.33/0.35			
11q23.1	c11orf92	rs3087967	0.24/0.39	0.44	0.002^a^	1.22 (1.07, 1.38)
	POU2AF1	**rs3802842**	0.24/0.39			
12q13.13	DIP2B	**rs7136702**	0.39/0.51	0.4	0.249	1.08 (0.95, 1.22)
	ATF1					
15q13.3	GREM1	**rs4779584**	0.17/0.82	0.81	0.5944	1.14 (0.94, 1.38)
16q22.1	CDH1	*rs2059254*	0.29/0.19	0.21	0.052	0.86 (0.73, 1.00)
		**rs9929218**	0.29/0.20			
18q21.1	SMAD7	**rs4939827**	0.47/0.24			
		rs7226855	0.47/0.25	0.33	0.0687	1.13 (0.99, 1.28)
20p12.3	FERMT1	**rs961253**	0.35/0.08			
	BMP2	rs5005940	0.40/0.07	0.07	0.976	1.00 (0.79, 1.28)

CEPH Europeans (CEU)-identified index and surrogate SNPs in bold and italics font respectively.

Singapore Chinese (SCH) surrogate SNPs that are different from the CEU i/sSNPs in normal font.

^1^Minor allele frequencies (Caucasian/Chinese Han Beijing) from HapMap Release 28.

^2^Note: Minor allele in CEU maybe major allele in SCH/CHB.

^a^Significant after Bonferroni correction (P<0.0031).

SNP locations based on Human Genome build 36.

### Statistical Analysis

The CHP (genotypes) files from the genome-wide scan were imported into Golden Helix SVS for statistical analysis. SNP loci that were not in Hardy-Weinberg Equilibrium (p≤1E-7) in the controls were filtered out. Principal component analysis (PCA) was performed on 869, 371 autosomal SNPs on all 2,000 samples and 270 HapMap (consisting of 90 CEU, 45 Chinese Han Beijing (CHB), 45 Japanese (JPT) and 90 Yoruba (YRB)) and 268 Singapore Genome Variant Project (SGVP) samples. The cases and controls clustered with the CHB and the SGVP Chinese samples indicating that there is no population substructure. Sixteen outliers, including two controls that probably have admixture ancestry, were removed. There was observable difference in the clustering of cases and controls for PC1. This difference was no longer apparent after PC1 correction (Figure S1).

Since the hypothesis tested in this study was whether the CEU-identified SNPs for CRC risk can be replicated in the SCH, the multiple testing corrections included only the number of at-risk SNPs investigated. Thus, a Bonferroni correction of 0.0031 (0.05/16) was applied. Multivariate logistic regression using the additive model was performed after adjusting for PC1. SNPs with p<0.0031 or 0.0031<p<0.1 were considered to be significantly or showed a trend of being associated with disease risk respectively. Subgroup analysis was performed for selected SNPs. The iSNPs were examined whenever possible. If the iSNP was not found on the SNP 6.0 platform or was non-polymorphic in SCH (MAF<0.01), surrogate SNP (sSNP) in high L.D. (r^2^>0.8) and within 100 kb vicinity of iSNP was identified from CHB individuals from HapMap and examined. sSNPs that were recently identified by fine mapping in CEU were interrogated whenever possible [2], [3]. The mean call rate of the eleven iSNPs and sSNPs interrogated was 0.99 (ranging from 0.97 to 1) and the genotypes of these SNPs clustered well.

Recurrence was defined as time from operation to local recurrence and/or distant metastasis. All patients without recurrence up till January 31^st^ 2012 were censored. Kaplan-Meier analysis with log rank test was used to evaluate the relationship between genotype and recurrence-free survival. Cox regression test was used to test the independence of the covariates and to estimate the risk for recurrence.

## Results and Discussion

There were 14% more males than females in this cohort (Table 1). Majority of the cases and controls were within the age range of 61–80. About 2/3 of the cases had colon cancer while early (Dukes A and B) and advanced (Dukes C and D) stages of CRC were almost equally represented. Most of the CRC cases were moderately differentiated. The clinico-pathological features of the cases were representative of the Singapore CRC patients.

Three candidate regions have either no iSNPs on the Affymetrix SNP6 platform (14q22 and 19q13.1) or the genotypes of iSNP rs4925386 (20q13.33) clustered poorly. All three regions have no sSNPs at high LD (r^2^≥0.8) within 100 kb of the iSNPs, as exemplified by the LD plot of chromosome 19q13.1 (Figure 1A). Thus, it is unlikely that these candidate regions harbor any SNP that could tag causal variant associated with CRC risk in SCH.

The only SNP out of the eleven interrogated that was significantly associated with CRC risk in SCH was sSNP rs3087967 at 11q23.1 (Figure 1B), possibly due to the higher minor allelic frequencies (MAF) and the relatively higher effect size (Table 2). Contrary to GWAS studies in Caucasians and Japanese [11], [12], we did not find this variant at 11q23.1 to be associated with greater disease risk in the rectum (OR = 1.20, 95% CI 1.02–1.42) compared to colon (OR = 1.22, 95% CI 1.06–1.41) (Table S1). We, however, found rs3087967 to be associated with greater CRC risk in men (OR = 1.34, 95% CI 1.14–1.58; p = 0.0005) compared to women (OR = 1.07, 95% CI: 0.88–1.29; p = 0.4954), thus implying a gender-specific role which has not been previously reported. It is interesting to note, however, that iSNP rs3802842 at 11q23.1 was replicated in the Northern Chinese but not the Hong Kong Chinese study [5], [6]. It is unclear why this so but the Hong Kong Chinese sampled could be a mixture of migrant workers from all over China as Hong Kong is a cosmopolitan city.

Five SNPs, rs6687758, rs11986063,, rs6983267, rs2059254, and rs7226855 at 1q41, 8q23.3, 8q24.21, 16q22.1 and 18q21.1 respectively show trend of association (0.0031<p<0.1) with CRC in SCH but have not reached statistical significance probably due to insufficient sample size and hence power (Table 2). The MAF for these 5 SNPs were also smaller than the CEU although the effect sizes were comparable.

The iSNP, rs6983267, at 8q24.21 was the first susceptible loci to be identified in the Caucasians [13], [14]. It was also the most frequently replicated iSNP in several different populations [5], [11], [15]–[17]. Interestingly, rs6983267 was reported to be significantly associated with CRC risk in both the Japanese and Northern Chinese in a recessive model only [5], [15]. We found rs6983267 to have higher effect size using a dominant model instead in SCH (OR = 1.38, 95% CI 1.13–1.69). It is unclear why this is so but the Japanese were found to be genetically closer to Northern Han Chinese than Southern Han Chinese [8]. The Hong Kong study, however, did not find rs6983267 but another SNP, rs7014346, at 8q24.21 to have evidence of association with CRC risk [6].

Further, sSNP rs827401 at 10p14 was associated with decreased cancer risk in rectum (OR = 0.74, 95% CI 0.63–0.88; p = 0.0006) but not colon (OR = 1.02, 95% CI 0.89–1.18; p = 0.7466) in SCH (Table S1), thus supporting earlier findings in the Caucasian and Northern Chinese [5], [18]. A recent study has reported that the iSNP at 10p14 was associated with a reduced risk of recurrence [19]. We, however, were not able to replicate this with sSNP rs827401 in our rectal cancer patients. Kaplan-Meier analysis revealed that the genotype was not significantly associated with recurrence-free survival either in all patients or patients stratified by chemotherapy. Patients with the protective allele (AB/BB) has hazard ratio of 0.91 (95% CI 0.69–1.20, p = 0.50) with AA as reference.

Notably, iSNPs rs7136702 (12q13.13) and rs4779584 (15q13.3) and sSNPs rs12638862 (3q26.2) and rs5005940 (20p12.3) did not show any evidence of being associated with CRC risk in SCH (Table 2; OR ∼1; p-value >0.1). The report on Northern Chinese found rs961253 at 20p12.3 to be significantly associated with CRC risk (OR = 1.38; 95% CI 1.19–1.60; p = 0.00002) [5]. We could not replicate this finding with sSNP rs5005940 (OR = 1.00; 95% CI 0.79–1.28; p = 0.976) although the LD structure in SCH is similar though not identical to the HapMap CHB samples (Figure 1C and 1D), suggesting that genetic heterogeneity exists between Northern and Southern Chinese. Similarly, rs4779584 at 15q13.13, with the risk allele being the major allele in the Chinese (Table 2), was replicated in the Hong Kong Chinese but not the Northern Chinese and SCH [5], [6].

**Table 3:** Comparison of effect sizes of CEU-identified variants for CRC across CEU and three different Chinese populations.

CEU#			NCH?		HK-CH^&^		SCH	
Chr	iSNP	OR (95% CI)	iSNP	OR (95% CI)	iSNP/sSNP	OR (95% CI)	iSNP/sSNP	OR (95% CI)
1q41	rs6687758	1.09 (1.06, 1.12)*					rs6695584	1.16 (0.98, 1.36)*
3q26.2	rs10936599	0.93 (0.91, 0.96)*					rs12638862	0.98 (0.87, 1.12)
8q23.3	rs16892766	1.25 (1.19, 1.32)*	rs16892766	NP			rs11986063	1.40 (1.04, 1.89)*
8q24.21	rs6983267	1.27 (1.16, 1.39)*	rs6983267	1.23 (1.13–1.34)*	rs7014346	1.23 (1.05–1.44)*	rs6983267	1.15 (1.00, 1.31)*
10p14	rs10795668	0.89 (0.86, 0.91)*	rs10795668	1.23 (1.12–1.34)**	rs10795668	1.28 (1.1–1.5)*	rs827401	0.74 (0.63, 0.88)**
11q23.1	rs3802842	1.11 (1.08, 1.15)*	rs3802842	1.29 (1.18–1.40)*	rs3802842	1.10 (0.95–1.27)	rs3087967	1.22 (1.07, 1.38)*
12q13.13	rs7136702	1.06 (1.04–1.08)*					rs7136702	1.08 (0.95, 1.22)
14q22	rs4444235	1.11 (1.08–1.15)*	rs4444235	1.08 (0.99–1.18)	rs4444235	1.02 (0.88–1.18)	rs4444235	NS
15q13.3	rs4779584	1.23 (1.14, 1.34)*	rs4779584	1.02 (0.92–1.13)	rs4779584	1.26 (1.04–1.52)*	rs4779584	1.14 (0.94, 1.38)
16q22.1	rs9929218	0.91 (0.89, 0.94)*	rs9929218	1.09 (0.97–1.23)	rs9929218	1.00 (0.84–1.18)	rs2059254	0.86 (0.73, 1.00)*
18q21.1	rs4939827	1.18 (1.12, 1.23)*	rs4939827	1.17 (1.05–1.31)*	rs4939827	1.17 (1.01–1.37)*	rs7226855	1.13 (0.99, 1.28)*
19q13.1	rs10411210	1.15 (1.10–1.20)*	rs10411210	1.10 (0.99–1.22)	rs10411210	1.02 (0.85–1.24)	rs10411210	NS
20p12.3	rs961253	1.12 (1.08, 1.16)*	rs961253	1.37 (1.19–1.59)*	rs355527	1.10 (0.84–1.45)	rs5005940	1.00 (0.79, 1.28)
20q13.33	rs4925386	0.93 (0.91,0.95)*					rs4925386	NS

# CEU SNP reference sources: as reported in the GWAS catalogue for CRC: http://www.genome.gov/gwastudies/index.cfm?pageid=26525384#searchForm.

?Northern Chinese (reference 5); ^&^Hong Kong Chinese (reference 6); NP =  non-polymorphic; NS =  no surrogate SNP at high LD within 100 kb of iSNP; empty cell  =  SNP not interrogated; *show evidence of being associated with CRC risk; **show evidence of being associated with rectal cancer risk.

In summary, only iSNPs or sSNPs at 1q41, 8q23.3, 8q24.21, 11q23.1, 16q22.1 and 18q21.1 showed evidence of association with CRC in SCH (Table 2). rs827401 at 10p14 was associated with increased risk in rectal cancer only. Moreover, in contrast to the findings of a recent study [19], the 10p14 region was not associated with disease prognosis in our series. Susceptibility loci from seven other candidate regions, 3q26.1, 12q13.13, 14q22, 15q13.3, 19q13.1, 20p12.3 and 20q13.3 showed no evidence of being associated with the disease. It is noteworthy that all four BMP loci, *BMP4* (14q22), *GREM1* (15q13.3), *BMP2* (20p12.3) and *LAMA 5* (20q13.33), the BMP pathway genes highlighted in a recent study [3], did not replicate in SCH. Chromosome 15q13.3 has been implicated to harbor the *CRAC1/HMPS* locus in Ashkenazi Jewish hereditary mixed polyposis syndrome (HMPS) patients [20]. We previously showed that the disease in Singapore Chinese HMPS patients was not linked to 15q13.3, and identified *BMPR1A* at 10q23 to be the disease-causing gene [21]. These earlier results indicate that genetic heterogeneity can give rise to similar clinical phenotypes in different populations.

Of the fourteen CEU-identified variants for CRC, only SNPs at 8q24.21, 10p14, 11q23.1 and 18q21.2 were replicated in at least two out of the three Chinese populations, suggesting that the functional variants in these regions could be important for colorectal tumorigenesis across diverse populations (Table 3). Amongst the four SNPs, only rs4939827 at 18q21.1 appear to tag a gene, SMAD 7, in the TGF-â signaling pathway, an important pathway in colorectal tumorigenesis [22]. The other three SNPs are in gene deserts. Accumulating evidence indicate that rs6983267 at 8q24.1 lies within a long range enhancer regulating the expression of C-MYC, an oncogene more than 300 kb downstream by binding T cell factor 4 (TCF4) and enhancing Wnt signaling [23]–[25]. Recent report has indicated, however, that there is neither somatic loss of the risk allele nor possible functional enhancer elements in the LD region at 10p14 and 11q23.1 [26], implying therefore that other unknown mechanisms may be responsible for the association.

In addition, the 8q23.3 region harboring the *EIF3H* and *UTP23* genes could be potentially important risk region for the Chinese as well, as sSNP rs11986063 was replicated with the highest effect size in SCH. The 8q23.3 region was not interrogated in the other two Chinese studies due to the lack of polymorphism in the iSNP rs16892766. Pittman et al showed that SNP rs16888589 at 8q23.3 bind EIF3H promoter and repressed its transcription [27]. A later eQTL expression analysis indicated however that the expression of UTP23, rather than that of EIF3H, was correlated with the risk allele of rs16888589 at 8q23.3. The authors suggested that both genes could be functionally coordinated [2].

Not all CEU-identified variants were replicated in the Chinese. The disparity could be due to differences in allelic frequencies and LD structures or real genetic differences. Since the effect sizes of these variants are relatively small and a recent study has estimated that at least 60 common variants contribute to CRC risk [28], the results imply that other variants contributing to predisposition to CRC remained to be identified.

## Supporting Information

Figure S1 **PCA plots for PC1 vs PC2 and PC2 vs PC3.**
(DOC)

Methods S1 **DNA Extraction from buffy coat and normal mucosa.**
(DOC)

Table S1 **Association of risk SNPs with tumor site.**
(DOC)
