# Supplementary material for: Association of Caucasian-Identified Variants with Colorectal Cancer Risk in Singapore Chinese
Source: PLoS One. 2012 Aug 3;7(8):e42407. doi: 10.1371/journal.pone.0042407 (PMC3411754; doi:10.1371/journal.pone.0042407)
Supplement: Figure S1 — PCA plots for PC1 vs PC2 and PC2 vs PC3. (DOC) [file pone.0042407.s001.doc]

**
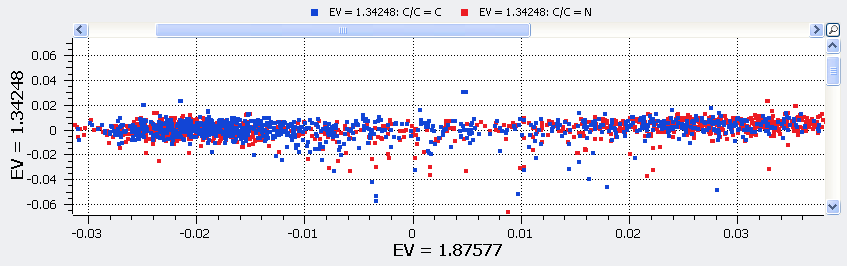
**

PC2

PC1

**
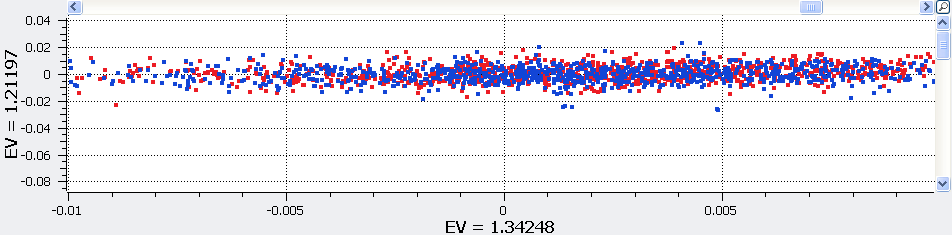
**

PC3

PC2

Figure S1: PCA plots for PC1 vs PC2 and PC2 vs PC3. Blue and red dots represent cases and controls respectively.
