# Supplementary material for: Association of Caucasian-Identified Variants with Colorectal Cancer Risk in Singapore Chinese
Source: PLoS One. 2012 Aug 3;7(8):e42407. doi: 10.1371/journal.pone.0042407 (PMC3411754; doi:10.1371/journal.pone.0042407)
Supplement: Methods S1 — DNA Extraction from buffy coat and normal mucosa. (DOC) [file pone.0042407.s002.doc]

# Supplementary Methods

# DNA Extraction from buffy coat and normal mucosa.

The white blood cells were pelleted from the buffy coat with cold erythrocyte lysis buffer. Normal mucosa tissue was frozen in liquid nitrogen and homogenized in cold nucleic lysis buffer. After this step, DNA extraction protocol is similar for both types of cells. Cells were lysed in 10% SDS and 20mg/ml of Proteinase K at 55C till ‘clear’ (at least 5 h) and DNA extracted by vortexing in 6M NaCl for 15s. DNA was precipitated from the supernatant in cold absolute ethanol and washed in 70% ethanol. DNA pellet was re-suspended in reduced EDTA TE buffer and incubated at 55C overnight for complete dissolution.
