# Supplementary material for: Association of Caucasian-Identified Variants with Colorectal Cancer Risk in Singapore Chinese
Source: PLoS One. 2012 Aug 3;7(8):e42407. doi: 10.1371/journal.pone.0042407 (PMC3411754; doi:10.1371/journal.pone.0042407)
Supplement: Table S1 — Association of risk SNPs with tumor site. (DOC) [file pone.0042407.s003.doc]

| **Table S1: Association of risk SNPs with tumor site** | | | | | | |
| --- | --- | --- | --- | --- | --- | --- |
| **Genotype** | | **No. of controls** | **Tumor Site** | | | |
|  |  |  | **Colon** | | **Rectum** | |
|  |  |  | **No. of cases** | **OR (95% Cl)** | **No. of cases** | **OR (95% Cl)** |
|  |  |  |  |  |  |  |
| **rs827401** | CC | 291 | 179 | 1.00 | 138 | 1.00 |
| **(10p14)** | CT/TT | 701 | 448 | 1.02 (0.89, 1.18) | 224 | 0.74 (0.63, 0.88) |
|  | P^†^ |  | 0.002* | | | |
|  |  |  |  |  |  |  |
| **rs3087967** | GG | 321 | 160 | 1.00 | 99 | 1.00 |
| **(11q23.1)** | AG/AA | 671 | 468 | 1.22 (1.06, 1.41) | 264 | 1.20 (1.02, 1.42) |
|  | P^†^ |  | 0.883 | | | |

| ^†^ Test for heterogeneity |
| --- |
| * Significant at p<0.1 |
